# Use of ABTHERA™ for an extensive abdominal wall defect caused by entrapment in a noodle stirring machine: a case report

**DOI:** 10.1016/j.tcr.2024.101058

**Published:** 2024-06-06

**Authors:** Yoshitaka Ooya, Shuji Takahira

**Affiliations:** Department of Trauma and Emergency Acute Medicine, Saitama Medical University International Medical Center, Saitama-Pref, Japan

**Keywords:** Abdominal wall injury, Open abdominal management, Abdominal wall reconstruction

## Abstract

An extensive abdominal wall defect is rare but severe trauma. Here, we have described the case of a male patient in his 20s who sustained extensive abdominal wall injury and intra-abdominal organ damage after being caught in a noodle stirring machine. We used ABTHERA as a substitute for a defective abdominal wall, achieved open abdominal management and temporary closure of a wide abdominal wall defect, and performed staged reconstruction surgery.

## Introduction

After an abdominal injury, the open abdomen must be managed during damage control surgery and for abdominal compartment syndrome. In this report, we have described a case of severe abdominal trauma caused by a noodle stirring machine that resulted in an extensive abdominal wall defect. Open abdominal management (OAM) was achieved using an ABTHERA™ Dressing Kit, and abdominal wall reconstruction with skin flap surgery was performed.

## Case presentation

A male in his 20s was injured when his abdomen became entangled in the shaft of a noodle stirrer while working at a noodle factory. The abdominal wall suffered an injury by the shaft of the agitator, and the intestinal tract had prolapsed, which made shaft removal difficult. A physician was sent to the location, where the agitator was disassembled and first aid was provided simultaneously. The patient was sent to the hospital with the shaft entangled in his abdomen.

**Fig. 1:**
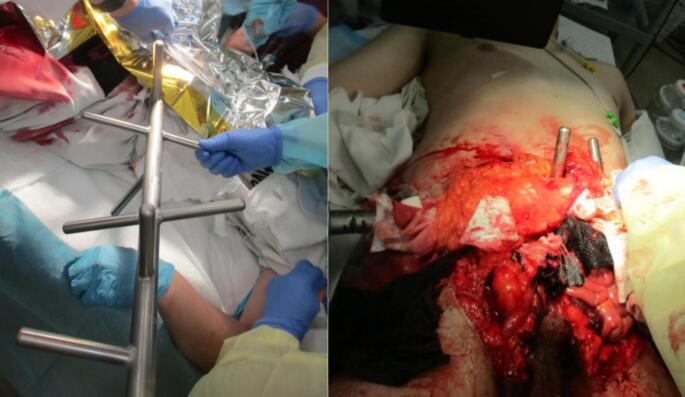
a. Agitator shaft in the abdominal wall. b. Extensive abdominal wall defect and prolapsed intestine.

Examination at the time of hospital admission revealed the following: Glasgow Coma Scale, E4V5M6; blood pressure, 145/102 mmHg; pulse, 128 beats/min; respiratory rate, 24 breaths/min; body temperature, 35.7 °C; and SpO_2_, 98 % (room air). The patient was slightly disturbed with marked cold sweats. Abdominal examination showed intestinal evacuation with an agitator shaft trapped in the abdomen (Fig. 1a, b).

The patient underwent surgery immediately upon arrival at the hospital, and the trapped clothing and shaft were removed from the abdomen. The abdominal wall was torn in a complex manner and was difficult to repair. However, the intra-abdominal organ damage was repaired, and the patient was admitted to the intensive care unit with OAM using ABTHERA™. On days 2, 4, and 7, the patient underwent the second, third, and fourth surgeries to clean the abdomen, replace the ABTHERA™, and resect the necrotic abdominal wall, respectively. On day 8, the patient's general condition was stable, and he was extubated and weaned from the ventilator. On day 10, the patient underwent left lateral femoral flap surgery by a plastic surgeon, and continuous negative pressure wet therapy (NPWT) was initiated. The patient's general condition stabilized postoperatively, and his activities of daily living improved. The patient was transferred to the Department of Plastic Surgery for further wound management on day 62 of hospitalization due to partial necrosis of the flap site. Conservative management was continued, and the patient was discharged on day 87.

Even after 3 years of injury, the patient has a huge hernial gate on the right side of his abdomen; however, he is in good general condition and has returned to work as a driver in the transportation industry.

## Intraoperative findings and surgeries

The abdominal wall was severely ruptured. The serosa of the small intestine was damaged and perforated 80 and 120 cm, respectively, from the ligament of traits, and the mesentery of the small intestine was damaged 130 cm from the ligament of traits. The right external iliac artery was ruptured. The abdominal wall, including the right rectus abdominis muscle, was severely damaged and was expected to be a source of infection due to a lack of blood flow. Partial resection and reconstruction of the injured small bowel were achieved using functional end-to-end anastomosis. After thrombectomy, an artificial graft was placed in the ruptured right external iliac artery. The abdominal wall was resected (10 × 10 cm), where necrosis was likely to occur because of severe damage. OAM was performed using ABTHERA™.

**Fig. 2:**
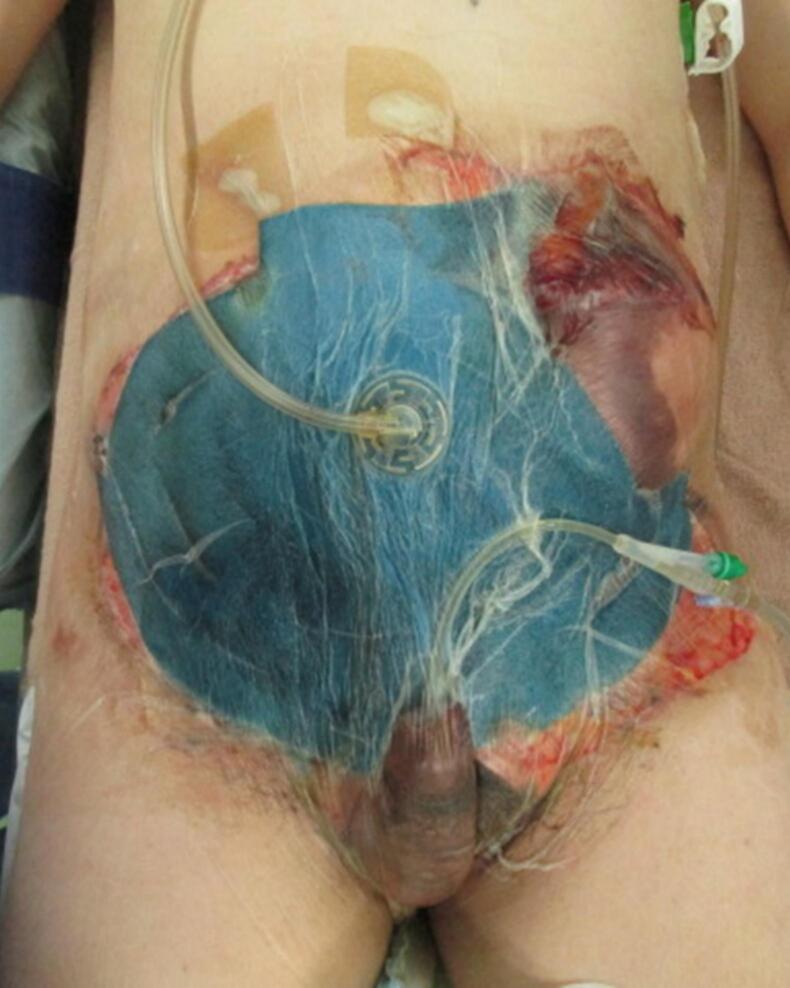
Temporary abdominal closure with ABTHERA after the second surgery.

**Fig. 3:**
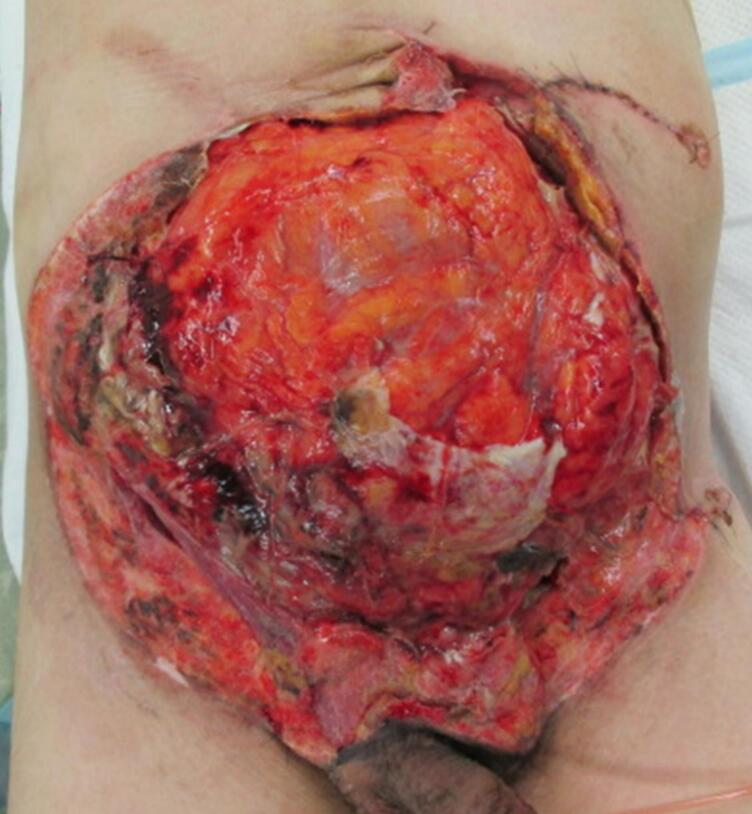
Covering of the intra-abdominal organs with biofilm during the fourth surgery.

**Fig. 4:**
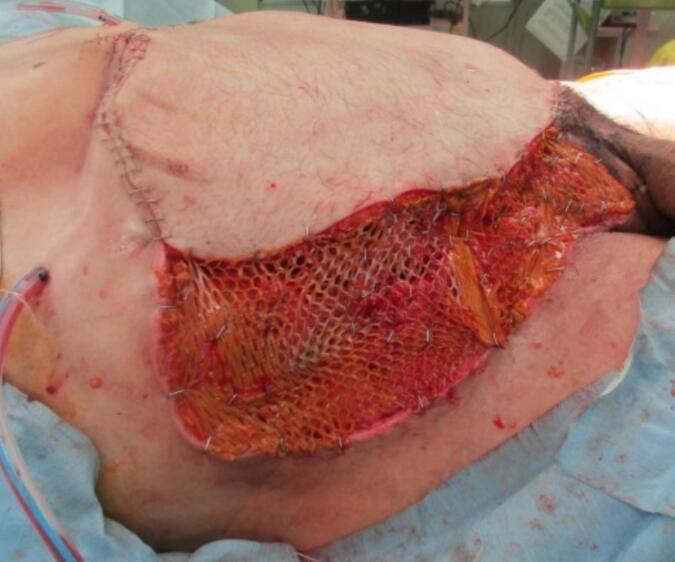
3× mesh segmented skin graft (anterior side) and skin flap from left lateral femur (posterior side).

**Fig. 5:**
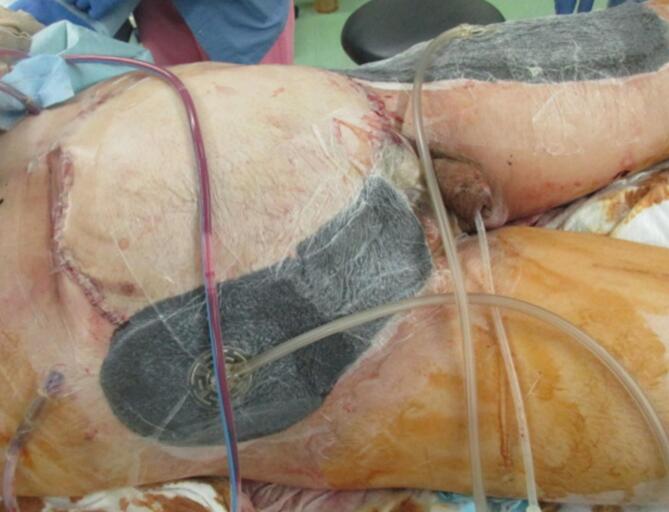
Postoperative skin valvuloplasty. NPWT was used to manage the skin flap extraction area and segmental skin graft area.

On the second day of hospitalization, another operation was performed to examine the abdomen. The small bowel anastomosis was clean, and no contamination or bleeding was detected in the abdominal cavity (Fig. 2). On the fourth day of hospitalization, the remaining abdominal wall tissue was debrided because of poor coloration. The torn skin was sutured as much as possible, and OAM with ABTHERA™ was continued. On the seventh day of hospitalization, the intestinal tract was covered with a large mesh and adhesions were observed. No infection or bleeding was detected (Fig. 3). OAM was continued with ABTHERA™. The abdominal cavity was in a good condition to allow wound closure. On the tenth day of hospitalization, a plastic surgeon created a 30 × 22-cm skin flap on the anterolateral side of the left thigh. Sternotomy was performed to reconstruct the abdominal wall. The skin defect was simultaneously treated with a segmental skin graft (Fig. 4, Fig. 5). The final diagnosis was extensive abdominal wall injury, right common iliac artery dissection, small bowel injury, and mesenteric injury.

## Discussion

Dennis et al. published a grade classification of abdominal wall injuries after blunt trauma. Most abdominal injuries are grades I–II, and only a few cases involve abdominal wall muscle damage [1]. Our case was the most severe grade VI, which is very rare. Only a few case reports have described severe grade VI abdominal wall injuries [2,3].

Damage control surgery is the treatment strategy for severe trauma. Damage control surgery consists of three phases: surgery to control bleeding and contamination, intensive care management to correct physiological abnormalities, and planned surgery for radical treatment [4]. OAM is often used for temporary abdominal closure after the initial surgery. OAM allows repeated surgical manipulations, including intraperitoneal lavage, to control infection because the abdomen is not closed. However, performing radical treatment as early as possible is crucial because complications, including fluid, protein, and electrolyte loss; infection; and gastrointestinal fistula, may increase with prolonged open abdomen. Among the various methods available for temporary wound closure in OAM, NPWT is the most effective [[5], [6], [7]]. NPWT is commonly used in the management of traumatic soft tissue defects, burns, necrotizing fasciitis, and post-amputation wounds. NPWT promotes granulation tissue formation and reduces infection when the wound is managed under negative pressure. Moreover, ABTHERA™ Dressing Kit is useful in fluid management because it protects the abdominal viscera, prevents external contamination, removes effusions and contaminated intra-abdominal fluid, and allows measurement of drainage volume [8,9].

The treatment of extensive abdominal wall injuries is difficult because of the lack of tissue for reconstruction, extensive organ damage, and the risk of infection. The treatment strategy is to initially protect exposed intra-abdominal organs and stabilize them hemodynamically, followed by the reconstruction of the abdominal wall once the patient stabilizes. Stone et al. advocated the following principles for the treatment of extensive abdominal wall defects: (1) avoid tension sutures and use mesh or other artificial materials, (2) avoid gastrointestinal anastomosis and create a colostomy if infection is present, and (3) perform the final abdominal wall reconstruction after closure of the gastrointestinal fistula [10].

In our case, the patient required OAM because of severe trauma, and most of the abdominal wall tissue required for abdominal wall closure was missing. Thus, temporary closure with autologous tissue was difficult. Although the patient's general condition stabilized after the initial surgery, the risk of intra-abdominal infection was high because of the open abdominal wall, repair of the gastrointestinal tract injury, and artificial blood vessel replacement. After the inflammatory response improved, the intra-abdominal organs were covered with a biofilm coating and the abdomen was closed in stages by performing a palpable skin flap procedure using the left rectus femoris muscle and a segmental layer skin graft.

In the past, abdominal wall closure was performed when single-stage abdominal wall reconstruction after OAM was difficult using mesh, anterior rectus sheath inversion, or fascial closure with Wittmann patch; however, the closure is difficult in cases with extensive abdominal wall defects, such as the present one.

## Funding

No funding was received for this study.

## CRediT authorship contribution statement

**Yoshitaka Ooya:** Writing – review & editing, Writing – original draft, Visualization, Validation, Supervision, Resources, Project administration, Methodology, Investigation, Formal analysis, Data curation, Conceptualization. **Shuji Takahira:** Investigation, Conceptualization.

## Declaration of competing interest

All authors have no conflict of interest with any organizations.
